# Exploration of health inequalities in patients treated with home non-invasive ventilation - Associations with respiratory healthcare burden

**DOI:** 10.1177/14799731261425236

**Published:** 2026-02-28

**Authors:** Wiktoria N. Milczanowska, Achaya Rajkumar, Nicholas J. Williamson, Oluwadamilare Falade, Laura Elliott, Amit S. Patel, Kai K. Lee

**Affiliations:** 1 GKT School of Medical Education, 405987 King’s College London, London, UK; 2 Department of Physiotherapy, 8948 King’s College Hospital NHS Foundation Trust, London, UK; 3 Department of Respiratory Medicine, 8948 King’s College Hospital NHS Foundation Trust, London, UK

**Keywords:** home-NIV, non-invasive ventilation, health inequalities, chronic respiratory failure, respiratory healthcare burden

## Abstract

**Background:**

Health inequalities affect many respiratory diseases. However, little is known about the extent or impact amongst patients treated with home non-invasive ventilation (NIV). This study explored health inequalities faced by these patients and associations with respiratory healthcare burden.

**Methods:**

A retrospective cohort study was conducted on patients actively receiving home-NIV treatment. Data on patient demographics, hospital healthcare burden and NIV adherence was collected between 4 October 2021 to 4 October 2023, and their relationships were evaluated.

**Results:**

187 patients met the inclusion criteria. Total hospital bed days was higher in females than in males, (11.7 ± 27.0 days vs 5.2 ± 12.5 days, *p* = 0.039), and increasing age was positively associated with higher number of respiratory-related hospital admissions (r = 0.146, *p* = 0.048). There was a weak correlation between deprivation rank and number of NIV care appointments missed (r = −0.163, *p* = 0.031). A higher BMI (>40 kg/m^2^) was associated with lower daily home-NIV use (68.7% ± 4.9% vs 83.0% ± 3.1% nights NIV used, *p* = 0.012).

**Conclusion:**

Patients with higher BMI had lower NIV adherence, females required more hospital bed days, older patients had more hospital admissions, and more deprived patients missed more hospital appointments.

## Introduction

Chronic respiratory diseases are a leading cause of death and disability worldwide, affecting over 450 million people globally.^1^ These diseases, which include obstructive sleep apnoea (OSA), obesity hypoventilation syndrome (OHS) and chronic obstructive pulmonary disease (COPD), can lead to chronic respiratory failure (CRF).^2^ Long-term home non-invasive ventilation (home-NIV) support is offered to individuals with CRF and requires regular specialist physician and multidisciplinary review.^3^

People from socioeconomically disadvantaged groups and marginalised communities experience a higher incidence and mortality rates of respiratory disease compared to the general population.^4^ These populations have disproportionally greater exposure to risk factors for respiratory disease (e.g., smoking, obesity), higher prevalence of multimorbidity, and barriers to accessing health services.^4^ Mistrust of healthcare organisations and public institutions may persist due to past and present systemic racism and discrimination towards underrepresented groups, contributing to health disparities such as reduced life expectancy.^5^ These issues have been contended as contributors to the heightened discrepancies seen during the COVID-19 pandemic.^5^ The result is a vicious cycle of systemic, avoidable, and unjust health inequality, defined as systematic, avoidable, and unjust differences in health outcomes (such as life expectancy, disease burden, and access to care) between population groups.^6^

Identification and reduction of health inequity is a key public health priority.^7^ However, little research has explored the impact specifically for patients treated with home-NIV, a group of patients who may face barriers to accessing respiratory care due to major symptom burden and high healthcare needs, leaving a potentially overlooked and neglected minority.^8^

We sought to explore for health inequalities amongst patients on home-NIV by assessing differences in treatment adherence and hospital healthcare burden between patient groups stratified by demographic and socioeconomic background.

## Methods

### Study design and population

A retrospective, single centre analysis was conducted at a specialist home ventilation centre (London, United Kingdom) as part of a service evaluation. Records of patients over the age of 18 years and registered as actively receiving domiciliary non-invasive ventilation (NIV) in the 2 years prior to 4^th^ October 2023 were screened. In order to minimise bias, records of all potentially eligible patients were screened. Patients who were found to have died, whose care had been transferred to another hospital, or had incomplete data in that period were excluded from the study, due to insufficient long-term data for the outcome analyses. Approval was granted by the local clinical governance office. The STROBE cohort checklist was used to write this report.^9^

### Data source and variables

Data was collected using hospital electronic patient record databases. Patient demographics collected included: age, sex, ethnicity, body mass index (BMI), comorbidities, housebound status, smoking status, indication for home-NIV, and the duration of home-NIV treatment. Self-reported ethnicities were categorised in accordance with ethnic group classification 6a from the Office for National Statistics.^10^ Deprivation status was assessed using the English Index of Multiple Deprivation (IMD), with deprivation rank grouped into deciles from 1 (most deprived) to 10 (least deprived).^11^

NIV adherence was quantified as (1) percent of days NIV used and (2) average nightly hours of use. Hospital healthcare burden was measured as the number of both emergency-related hospital presentations and admissions and non-emergency planned routine hospital visits, taken from hospital episode records. Emergency events were only counted if they were respiratory related concerns. Emergency parameters measured included; (1) Emergency Department (ED) attendances; (2) hospital admissions; and (3) total hospital bed days. Non-emergency visits quantified as; (1) total hospital appointments; (2) hospital appointments attended; (3) hospital appointments unattended.

### Statistical analysis

Statistical analysis was performed using Stata (Version SE 17.0). Continuous variables were summarised as mean ± SD or median (IQR) depending on the distribution of the data. Between-group comparisons were analysed using t-tests or ANOVA for normally distributed data and Mann–Whitney U or Kruskal–Wallis tests for non-normal data. Correlations were assessed using Pearson or Spearman coefficients as appropriate. Subgroup analysis by aetiology (OSA/OHS vs other) was conducted using chi-square and logistic regression. Missing data was handled by pairwise deletion, excluding cases from particular analyses where the relevant data was missing. Emergency episode data was available for 185 patients, hospital episode data for 178 patients, NIV adherence hours data for 158 patients, and NIV adherence % days used for 140 patients. A significance level of *p* < 0.05 was adopted throughout.

## Results

### Baseline characteristics

**Figure 1.**
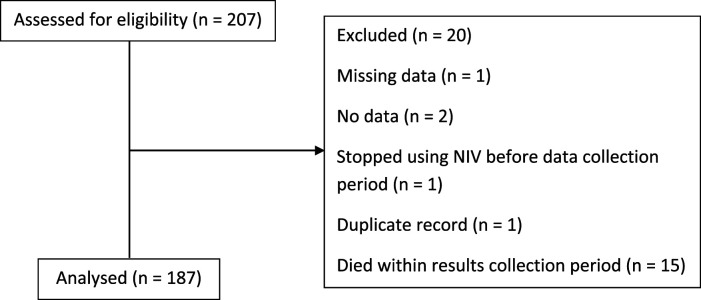
Flow diagram of participants.

Two hundred and seven patient records were identified and screened, of which 187 met the inclusion criteria (Figure 1). Patient characteristic data are summarised in Table 1. The mean age was 63 years, with 94 patients (50.3%) being male. Most patients were of White (*n* = 77, 41.2%) or Black (*n* = 64, 34.2%) ethnic origin. Statistical analysis of ethnicity only included the two most representative groups, Black and White. Comorbidities were common, with cardiovascular disease being the most prevalent (*n* = 73, 39.0%), followed closely by diabetes mellitus (*n* = 71, 38.0%). Most of the cohort (*n* = 152, 86.9%) were overweight (BMI > 25 kg/m^2^), with an average BMI of 40 kg/m^2^. Over half of patients (*n* = 106, 56.7%) were current or previous smokers. 20 patients (10.7%) were housebound.

**Table 1.** Patient characteristics. Data presented as mean ± SD, median (IQR) or *n* (%).

Characteristic	*N* = 187
Age (years)	63 ± 14
Sex (Males)	94 (50.3%)
Ethnicity	
Asian	9 (4.8%)
Black	64 (34.2%)
White	77 (41.2%)
Other	10 (5.3%)
Not known	27 (14.5%)
Deprivation Decile (IMD)	3 (2-5)
1 (most deprived area)	3 (1.6%)
2	50 (26.9%)
3	46 (24.7%)
4	20 (10.8%)
5	22 (11.8%)
6	17 (9.1%)
7	9 (4.8%)
8	14 (7.5%)
9	4 (2.2%)
10 (least deprived area)	1 (0.5%)
BMI (kg/m2)	40 ± 13
Housebound status	20 (10.7%)
Smoking status	
Never	59 (31.5%)
Past	67 (35.8%)
Current	39 (20.9%)
Not known	22 (11.8%)
Comorbidities	
Cardiovascular disease	73 (39.0%)
Hypertension	73 (39.0%)
Chronic renal disease	21 (11.2%)
Active cancer	7 (3.7%)
Mental health disorder	34 (18.2%)
Diabetes mellitus	71 (38.0%)
Musculoskeletal	28 (15.0%)
Indication for NIV	
OSA/OHS	125 (66.8%)
COPD	18 (9.6%)
Overlap syndrome (OSA/OHS with COPD)	28 (15.0%)
Other	13 (7.0%)
Not known	3 (1.6%)
Duration of NIV (months)	43.6 (15.4 – 74.8)

The most common indication for home-NIV was OSA/OHS, accounting for 125 patients (66.8%), followed by overlap syndrome in 28 patients (15.0%) and COPD in 18 patients (9.6%). The duration of treatment varied significantly, with a median (IQR) of 43.6 months (15.4 – 74.8).

### NIV adherence

Mean ± SD home-NIV use was 77.5% ± 32.2% of days, with an average nightly use of 6.0 ± 3.0 h. Table 2 shows the difference between NIV adherence by patient characteristic.

**Table 2.** NIV adherence by patient characteristics. Data presented as mean ± SD.

	Percent of days used (%)	Average nightly NIV use (hours)
Sex		
Male	75.5 ± 33.5	6.2 ± 2.8
Female	79.8 ± 30.7	5.8 ± 3.1
Ethnicity		
Asian	95.2 ± 12.6	6.5 ± 2.0
Black	74.7 ± 32.0	5.8 ± 3.0
White	81.0 ± 31.3	6.2 ± 3.2
Other	61.9 ± 38.0	5.7 ± 3.4
Deprivation Decile		
1	98.5 ± 2.1	8.9 ± 2.6
2	74.2 ± 33.0	5.3 ± 3.2
3	72. 9 ± 36.7	5.8 ± 3.5
4	78.3 ± 29.1	6.6 ± 2.7
5	83.2 ± 26.1	6.7 ± 2.3
6	70.3 ± 39.1	5.7 ± 2.5
7	85.2 ± 21.3	6.3 ± 1.9
8	83.4 ± 26.9	7.1 ±2.9
9	100	7.4 ± 0.3
10	98.9	5.3
Smoking		
Never	78.5 ± 28.1	5.8 ± 2.6
Past	80.4 ± 30.7	6.4 ± 3.1
Current	69.0 ± 38.9	5.3 ± 3.4
Mental health		
Diagnosis	78.4 ± 31.9	5.7 ± 2.7
No diagnosis	77.4 ± 32.4	6.0 ± 3.0

**Figure 2.**
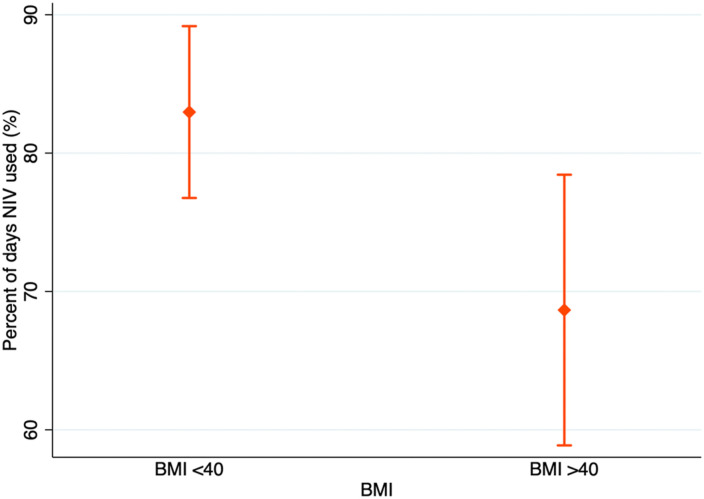
Comparison of daily NIV use (% of nights) in BMI groups.

Daily home-NIV use defined by percent of nights used was higher in patients with BMI <40 kg/m^2^ as compared to patients with BMI > 40 kg/m^2^ (83.0% ± 3.1% vs 68.7% ± 4.9%, *p* = 0.012) (Figure 2). However, the average hours of nightly NIV use were similar regardless of BMI above or below 40 kg/m^2^ (5.6 ± 0.4 h vs 6.1 ± 0.3 h, *p* = 0.342).

There was no relationship between either percent of nights with NIV use or average hours usage and age (Supplemental Table 1), sex, ethnicity, deprivation decile or smoking status (*p* = 0.098 to 0.812). NIV percent of nights used or average hours also did not differ between patients with and without documented mental health disorders (*p* = 0.890 and *p* = 0.630 respectively) (Table 2).

### Emergency attendances and hospital admissions

The mean ± SD number of ED attendances was 0.5 ± 1.1, respiratory-related hospital admissions was 0.5 ± 1.0 and total bed days was 8.5 ± 21.3 days, in the 2-year study period.

Table 3 and Supplemental Table 1 show the differences in ED attendances and respiratory-related hospital admissions by patient characteristic. There was a positive, significant association between hospital admissions and increasing age (r = 0.146, *p* = 0.048). No significant relationship was found between age and ED attendances (r = 0.142, *p* = 0.054) and total bed days (r = 0.127, p = 0.084). Hospital bed days were higher in females compared to males (11.7 ± 27.0 vs 5.2 ± 12.5 days, *p* = 0.039). There was no significant relationship between sex and ED attendances (*p* = 0.436) or hospital admissions (*p* = 0.416). Sub-group analysis by aetiology revealed that patients without OSA/OHS (*n* = 34) were significantly more likely to present to the emergency department with a respiratory-related illness during the study period than those with OSA/OHS (*n* = 153) (1.0 vs 0.4 attendances, *p* = 0.001).

**Table 3.** Correlation coefficients between age and BMI and emergency attendances and admissions. * = *p* < 0.05.

	ED attendances (number)	Hospital admissions (number)	Total bed days (number)
Age	0.142	0.146*	0.127
BMI	−0.08	−0.07	0.01

There was no relationship between BMI, ethnicity, deprivation decile, smoking status, or the presence of a mental health disorder and ED attendances, hospital admissions or total bed days rate (*p* = 0.143 to 0.925) (Supplemental Table 2).

### Non-emergency visits

The average number of hospital appointments in the 2-year study period for this cohort was 6.2 ± 4.0, of which 5.6 ± 3.8 were attended and 0.6 ± 1.2 were unattended.

Table 4 shows the difference in healthcare utilisation when analysed against patient characteristic. There was a weak negative correlation between deprivation decile and number of NIV care appointments missed (r = −0.163, *p* = 0.031), meaning that more deprived status was associated with more missed appointments. However, there was no relationship between deprivation status and total hospital appointments (*p* = 0.753), or number of appointments attended (*p* = 0.760). There was also no relationship between age, BMI, sex, ethnicity, smoking status and the presence of a mental health disorder and total hospital appointments, number of appointments attended and number of appointments unattended (*p* = 0.120 to 0.962).

**Table 4.** Non-emergency visits by patient characteristics. Data presented as mean ± SD.

	Total hospital appointments (number)	Hospital appointments attended (number)	Hospital appointments unattended (number)
Sex			
Male	6.5 ± 4.1	5.9 ± 3.8	0.6 ± 1.2
Female	6.0 ± 3.9	5.3 ± 3.8	0.6 ± 1.2
Ethnicity			
Asian	5.8 ± 4.5	5.5 ± 4.4	0.3 ± 0.5
Black	6.8 ± 4.0	6.0 ± 3.8	0.8 ± 1.2
White	6.1 ± 4.2	5.3 ± 3.9	0.8 ±1.3
Other	7.5 ± 4.2	7.0 ± 4.4	0.4 ± 0.4
Deprivation decile			
1	5.5 ± 6.4	0	0.5 ± 0.7
2	6.2 ± 4.1	1.6 ± 3.9	0.8 ± 1.2
3	6.6 ± 4.8	1.7 ± 4.3	1.0 ± 1.7
4	5.3 ± 4.4	1.8 ± 4.2	0.2 ± 0.5
5	6.7 ± 4.0	3.0 ± 4.0	0.5 ± 0.8
6	6.3 ± 2.5	0	0.3 ± 0.6
7	6.8 ± 2.5	1.1 ± 2.5	0.3 ± 0.5
8	6.3 ± 4.0	2.5 ± 4.2	0.5 ± 0.8
9	3.8 ± 0.5	3.8 ± 0.5	0
10	4.0	4.0	0
Smoking			
Never	6.4 ± 4.0	5.9 ± 3.6	0.5 ± 0.8
Past	6.4 ± 3.7	5.7 ± 3.6	0.7 ± 1.1
Current	5.6 ± 4.0	4.9 ± 4.2	0.7 ± 1.5
Mental health			
Diagnosis	5.9 ± 4.0	5.0 ± 4.1	0.9 ± 1.5
No diagnosis	6.3 ± 4.0	5.7 ± 3.7	0.6 ± 1.1

## Discussion

### Summary of main findings

Respiratory-related admissions for females were significantly longer, measured by total bed days, than for males (Supplemental Table 2). Female patients spent an average of 12 days in hospital compared to 5 days for males. However, no significant difference was found between sexes in the number of ED attendances or hospital admissions. This suggests that while exacerbation frequency may have been similar, the severity or complexity of episodes in females could have differed. Age was unlikely to be a contributing factor, as there was no significant age difference between male and female patients. Further analysis by disease aetiology did not reveal any sex-based differences. Additionally, sex-based delays in accessing emergency healthcare have previously been described and could have contributed.^12^ No further associations between sex and other variables were identified.

Increasing age was positively associated with number of respiratory-related hospital admissions. A plausible explanation could be the presence of multi-morbidity and the compounding nature of these pathologies on respiratory health, and many studies show impact of age on admission rates in respiratory disease.^13^ No further associations between age and other variables were identified.

Patients with a BMI greater than 40 kg/m^2^ had a lower daily NIV usage (68.7% ± 4.9% vs 83.0% ± 3.1%, *p* = 0.012). As expected, the most common underlying aetiology among patients with BMI > 40 kg/m^2^ was OSA/OHS. This may explain the lower adherence in this subgroup, as lower adherence levels have been reported in OHS as compared with COPD and other aetiologies.^14^ This may seem surprising, as the evidence is clear that sufficient daily use of NIV is critical for improving physiological outcomes and health-related quality of life.^15^ Nevertheless, the NIV usage levels in our study are comparable to adherence levels reported in OHS NIV trials.^16^ A multitude of factors can influence treatment adherence, and whilst it is beyond the scope of this study to postulate reasons from our data, a few often cited include underlying disease severity, perception of need and clinical benefit, and psychological reasons.^17^ No further associations between BMI and other variables were identified.

More deprived patients missed more NIV hospital appointments than less deprived patients (*p* = 0.031). This finding aligns with existing research on health inequalities, which consistently demonstrates that individuals in more deprived areas experience greater barriers to healthcare access due to a variety of factors, including income, transport and education levels.^5^ Clinically, these results highlight the need for targeted interventions to improve appointment adherence in more deprived communities, such as flexible scheduling or outreach programs. Future research should explore the underlying reasons for missed appointments in deprived areas and evaluate the effectiveness of tailored support strategies. No further associations between deprivation and other variables were identified.

Home NIV-usage data, with nightly adherence approaching 77.5% and an average nightly use of 6.0 h, was consistent with adherence levels reported in other studies.^18^ ED visits and hospital admission rates were low, with a mean admission rate of 0.5 episodes per year, comparable to rates observed in other cohorts with OHS.^16^ Sub-group analysis by aetiology revealed that patients without OSA/OHS (*n* = 34) were significantly more likely to present to the emergency department with a respiratory-related illness during the study period (*p* = 0.001). This finding may indicate that alternative indications for home-NIV are associated with more severe CRF or a higher risk of exacerbation. However, given the small size of this sub-group, these results should be interpreted with caution.

A notable negative finding was the absence of a difference in home-NIV adherence between patients with and without a documented mental health diagnosis, despite the expectation that psychological factors may influence treatment adherence.^17^ The prevalence of mental health diagnoses in this cohort (18.2%) was comparable to the estimated 22.6% reported in the UK general adult population in 2023/24.^19^ It is important to recognise that this retrospective analysis depended on the documentation of mental health conditions in hospital medical records; therefore, cases of undocumented, historic, or untreated mental health conditions may have been missed. Nevertheless, these findings suggest that home-NIV can be accepted and adhered to by patients with mental health diagnoses at similar levels to those without.

We did not find any associations between ethnicity and our study health outcomes, with all ethnic groups demonstrating similar distributions in NIV adherence and healthcare utilisation. Our results suggest that ethnicity may not be a major influence on NIV treatment adherence. To our knowledge, and at the time of writing, ethnicity-related differences in home-NIV treatment adherence has not previously been reported. However, ethnicity has consistently been shown to influence Continuous Positive Airway Pressure treatment adherence rates.^20^ Therefore, whilst our findings do not show differences and are therefore encouraging, we would advocate further studies to confirm this.

Our study population was diverse, with a range of respiratory comorbidities typical of home-NIV cohorts reported in previous studies.^18^ The proportion of past (35.8%) and current (20.9%) smokers aligns with similar home-NIV study populations.^21^ The ethnic composition of the cohort reflected the diversity of the communities served by the hospital.^22^ While patient factors and disease patterns may vary between regions, the predominance of sleep-disordered breathing in our home-NIV cohort matches studies elsewhere.^14,21^

It is important to recognise the intersectionality of the characteristics analysed. Deprivation, ethnicity, gender and other personal characteristics can collectively have a more profound impact on treatment adherence and hospital utilisation than any single characteristic alone. Factors traditionally associated with healthcare inequalities: smoking status, ethnicity, deprivation and presence of mental health disorder, did not significantly impact NIV adherence or hospital utilisation in our study. This could suggest equity in the provision of home NIV treatment, but could also indicate that other variables, such as disease severity, are overshadowing the influence of these characteristics. Future studies in larger sample sizes should explore the intersectionality of these characteristics.

### The strengths and limitations of this study

A key strength of this study was the exploratory nature of the dataset, offering a comprehensive overview of home-NIV patients and a broad range of variables linked to respiratory health inequalities identified in similar research. This context supported a meaningful comparison between patient characteristics and respiratory healthcare burden. Notably, the dataset had a balanced sex representation, addressing the common issue of underrepresentation of females in clinical research.^23^ Our sample size of 187 patients exceeds the typical cohort size for home ventilation centres, as reported in a European survey of home mechanical ventilation centres.^24^

Limitations of our study include the retrospective nature, with inherent biases such as reliance on medical records and limited ability to verify data accuracy. Additionally, we were unable to study other important potential variables, including characteristics such as occupation, religion, education, social capital, health literacy, barriers to accessing healthcare services, and air pollution exposure. However, this approach provides a real-world dataset that complements the controlled conditions of clinical trials, and our study method and measures were defined a-priori to minimise risk of observer bias. We were able to complete a 2-year observation period, which can be more challenging with prospective studies, however even with this study duration we observed low event rates for some outcomes such as hospital admission rates; future studies should consider longer follow-up periods. Patients included were those actively receiving home-NIV during the study period; a more robust approach would have been to follow up patients from point NIV initiation, but this data was unavailable at the time. Our study period began some months after the final national lockdown following the COVID pandemic, whilst clinical services were returning to normal, patient behaviour could have been altered during this time. Finally, as this was a single-centre study, our findings may not be generalisable to settings with different demographic profiles or healthcare systems. Our results should be considered exploratory. Nevertheless, we hope this work will stimulate further studies to confirm the findings. Larger cohort studies would permit more detailed sub-group analyses and exploration of intersectionality of characteristics.

## Conclusions

This study aimed to explore if patient characteristics influence treatment adherence and respiratory healthcare burden in patients using home-NIV. Patients with higher BMI had lower NIV adherence, females required more hospital bed days, older patients had more hospital admissions, and more deprived patients missed more hospital appointments. This work highlights that health inequalities do exist amongst patients on home-NIV. Further studies are needed to confirm these findings in other populations and explore solutions for these social differences, helping to develop equitable frameworks to improve health outcomes for this severely affected and vulnerable group.

## Supplemental material


Supplemental material - Exploration of health inequalities in patients treated with home non-invasive ventilation - Associations with respiratory healthcare burden
Supplemental material for Exploration of health inequalities in patients treated with home non-invasive ventilation - Associations with respiratory healthcare burden by Wiktoria Milczanowska, Achaya Rajkumar, Nicholas J Williamson, Oluwadamilare Falade, Laura Elliott, Amit S Patel, Kai K Lee in Chronic Respiratory Disease

## Data Availability

Data is not publicly available but may be obtained upon reasonable request from the corresponding author.*
