# Supplementary material for: Exploration of health inequalities in patients treated with home non-invasive ventilation - Associations with respiratory healthcare burden
Source: Chron Respir Dis. 2026 Feb 28;23:14799731261425236. doi: 10.1177/14799731261425236 (PMC12954001; doi:10.1177/14799731261425236)
Supplement: Supplemental material - Exploration of health inequalities in patients treated with home non-invasive ventilation - Associations with respiratory healthcare burden [file sj-pdf-1-crd-10.1177_14799731261425236.pdf]

Supplementary Table 1. Correlation coefficients between age and BMI and NIV adherence. * = p<0.05

	Percent of days used (%)	Average nightly NIV use (hours)
**Age**	0.137	-0.019
**BMI**	-0.22*	-0.12

Supplementary Table 2. Emergency admissions by patient characteristics. Data presented as Mean ± SD.

	ED attendances	Hospital admissions following ED	Total hospital bed days
**Sex**			
Male	0.4 ± 1.1	0.5 ± 1.0	5.2 ± 12.5
Female	0.5 ± 1.1	0.6 ± 1.0	11.7 ± 27.0
**Ethnicity**			
Asian	0.4 ± 0.5	0.4 ± 0.5	14 ± 28.4
Black	0.5 ± 1.2	0.6 ± 1.2	8.3 ± 22.4
White	0.5 ± 0.9	0.5 ± 0.8	7.5 ± 19.0
Other	1.0 ± 2.5	0.8 ± 2.0	6.3 ± 18.9
**Deprivation Decile**			
1	0.3 ± 0.6	0.3 ± 0.6	18.7 ± 32.3
2	0.6 ± 1.0	0.6 ± 1.0	11.1 ± 23.0
3	0.5 ± 1.3	0.5 ± 1.3	4.0 ± 10.0
4	1.1 ± 1.7	1.1 ± 1.7	17.1 ± 19.6
5	0.2 ± 0.5	0.4 ± 0.7	7.5 ± 23.3
6	0.2 ± 0.4	0.2 ± 0.4	1.2 ± 3.2
7	0.2 ± 0.4	0.2 ± 0.4	1.7 ± 3.3
8	0.4 ± 1.1	0.4 ± 1.1	15.1 ± 36.2
9	0.5 ± 1.0	0.5 ± 1.0	8.5 ± 17
10	0	0	0
**Smoking**			
Never	0.4 ± 0.7	0.4 ± 0.8	8.2 ± 22.4
Past	0.7 ± 1.4	0.7 ± 1.3	12.5 ± 26.4
Current	0.4 ± 0.6	0.4 ± 0.7	5.2 ± 11.5
**Mental health**			
Diagnosis	0.5 ± 0.8	0.5 ± 0.8	9.0 ± 20.7
No diagnosis	0.5 ± 1.1	0.5 ± 1.1	8.4 ± 21.5
